# Frequency of seropositive celiac disease and hypothyroidism among children and adolescents with congenital heart disease: A case-control study

**DOI:** 10.1186/s12887-023-04283-9

**Published:** 2024-08-09

**Authors:** Mohammad Reza Edraki, Ali Jamshidi Kerachi, Negar Yazdani, Naser Honar, Reza Dehghani, Zhila Afshar, Mohsen Dehghani, Hamid Amoozgar

**Affiliations:** 1 Cardiovascular Research Center, Shiraz University of Medical Sciences, Shiraz, Iran; 2 School of Medicine, Shiraz University of Medical Sciences, Shiraz, Iran; 3 Community Based Psychiatric Care Research Center, School of Nursing and Midwifery, Shiraz University of Medical Sciences, Shiraz, Iran; 4 Department of Pediatric Gastroenterology, Neonatal Research Center, Shiraz University of Medical Sciences, Shiraz, Iran; 5 School of Medicine, Bam University of Medical Sciences, Bam, Iran; 6 Gastroenterohepatology Research Center, Nemazee Hospital, Shiraz University of Medical Sciences, Shiraz, Iran

**Keywords:** Child, Congenital heart defects, Hypothyroidism, Adolescence, Celiac Disease

## Abstract

**Background:**

The prevalence of celiac disease (CD) and hypothyroidism exhibit significant variation in different studies among patients with congenital heart disease (CHD). This study evaluated the frequency of laboratory test abnormalities in children and adolescents with CHD in Shiraz, Iran.

**Methods:**

This prospective case-control study was conducted on 223 children and adolescents with CHD and healthy individuals referred to the heart clinic affiliated with Shiraz University of Medical Sciences between February 2019 and December 2021. They were classified into case and control groups. Blood tests were performed for total IgA antibody, anti-tissue transglutaminase IgA antibody (anti-TTG Ab), T4, and thyroid stimulating hormone (TSH) and anti-thyroid peroxidase antibodies in serum, along with transthoracic echocardiography. Likewise, demographic characteristics of patients, including age, sex, weight, height, and body mass index (BMI), were recorded. Also, anti-TTG Ab levels were compared among CHD patients according to cyanosis status, gender, age (above and below five years), and BMI (under and over 18.5).

**Results:**

Ninety-eight CHD patients and 100 healthy individuals with an average age of 5.32 ± 4.05 years (1–18 years) were examined. In children with CHD, atrial septal defect (27%), ventricular septal defect (20%), and tetralogy of Fallot (13%) were the most prevalent disorders. Only one CHD patient had an anti-TTG Ab level of 16.6 unit/mL, considered borderline for seropositive CD diagnosis. There was no difference in anti-TTG Ab levels between age (above and below five years), BMI (under and over 18.5), cyanosis status, and gender groups. Seven CHD patients had high TSH levels, three had cyanotic CHD, and one had Down syndrome. The TSH levels and non-autoimmune hypothyroidism were significantly higher in CHD patients than in normal subjects (p < 0.05).

**Conclusions:**

According to the results of this study, the serum level of TSH and prevalence of non-autoimmune hypothyroidism were higher in patients with CHD than in normal subjects, but the serum level of anti-TTG Ab was not different between the two groups.

## Background

Celiac disease (CD) is an enteropathy linked to the immune system, defined as a permanent genetic sensitivity to wheat gliadin or other prolamins in oats [1]. This condition occurs through the interaction among gluten, immune factors, genetics, and environment [2]. The CD prevalence ranges from 0.5 to 3% worldwide [3]. For instance, its prevalence in Europe and the Asia-Pacific region is reported as 2.67%, while it is 1% in Iran [4].

Some studies support a possible association between congenital heart disease (CHD) and CD and thyroid dysfunction [5, 6]. However, a study by Bhargava et al. in India stated that the relationship between CD and CHD is rare [7].

The abnormal immunological profiles of the cellular and humoral immune systems in CD and thyroid disease have a genetic origin. Genetic ailments are also seen in CHDs [8], which may describe why the higher prevalence of some chromosomal disorders, such as Down syndrome, exists in CHD [4]. In some studies, CD was associated with several congenital malformations in the face, neck, ear, heart, and digestive system [5].

Furthermore, due to some genetic comparations, patients with CHD have a higher prevalence of thyroid dysfunction, so in some studies, 5% of infants with congenital hypothyroidism had CHD [9]. Among the origins of congenital hypothyroidism and CHD, we can mention the expression of the NKX2.5 genes in the heart and thyroid, its mutation, and the role of the HOXA3 and TBX1 genes in the development of the thyroid, blood vessels, and heart structures [10]. Also, during embryogenesis, the thyroid gland shares nuclear transcription factors with the heart and the great vessels during organogenesis [11]. Mild hypothyroidism has also been observed in about 12% of children with CHD, which is perhaps related to thyroid autoimmunity or dysgenesis [11]. One study reported a 10-fold higher risk of non-autoimmune hypothyroidism in CHD patients than in individuals without CHD [12]. However, there is still insufficient information about the prevalence and severity of thyroid disorders among patients with CHD.

Since the prevalence of CD and hypothyroidism in patients with CHD varies across different studies, this study aimed to examine the frequency of abnormal laboratory test results for these two conditions in children and adolescents with CHD in Shiraz, Iran.

## Methods

### Design and subjects

This prospective case-control study was conducted on children and adolescents diagnosed with CHD. They were referred to the heart clinic affiliated with Shiraz University of Medical Sciences between February 2019 and December 2021. A consecutive sampling method was followed. The study included children and adolescents aged 1 to 18 with cyanotic and non-cyanotic CHD, operated or not operated, and with or without Down syndrome. Among the heart diseases that can be classified according to the disease severity, patients with a moderate degree or more were included in this study. Patients with mild CHD grades, a deficiency of total serum IgA levels, a history of amiodarone prescription, previous multiple imaging procedures, or those who were ill or sick were excluded from the study. It should be noted that amiodarone and multiple X-ray imaging could interfere with the results of thyroid function tests.

### Measurements

In this study, CHD patients aged 1 to 18 years as the case group were compared with the corresponding healthy subjects as the control group. Based on valid echocardiography guidelines, CHD was diagnosed via transthoracic echocardiography using 2D, M-mode, doppler, and color doppler methods, and CHD patients were classified into two groups, cyanotic and non-cyanotic [13, 14].

We performed CD laboratory tests by measuring serum anti-tissue transglutaminase IgA antibody (anti-TTG Ab) and total IgA antibody. Five milliliters of blood were taken from the participants and stored at -20 °C for evaluation. The IgA level was measured by the indirect immunofluorescence method. The AESKU kit (number 3504) made in Germany was used to measure anti-TTG Ab, and the Pishtaz kit made in Iran was used to measure thyroid stimulating hormone (TSH) and T4. Both tests were performed on serum, and the sample was centrifuged at 60,000 rpm for 10 min. Anti-TTG Ab levels of more than 18 units/mL were categorized as positive, fewer than 16 units/mL as normal, and between these two as borderline.

Regarding the thyroid tests, the cut-off points for TSH, T4, and anti-thyroid peroxidase antibodies in our study were determined based on Table 1. The selection criterion for high TSH level was the level of our requested test or the patients’ previous tests (and of course only for three of them who were already taking levothyroxine) [15–17].

**Table 1:** Reference values for normal serum TSH, T4, and anti-thyroid peroxidase antibodies at selected ages

Variable	Age (years)	Normal level
TSH, 97.5 percentile (mU/L)	1–5	6.25
	6–10	5.40
	11–14	4.61
	15–18	4.33
T4, -2 SD (nmol/liter)	1–5	92.10
	6–10	73.56
	11–14	65.40
	15–18	62.27
Serum level of anti-TPO Ab	< 35 IU/ml	

_Anti−TPO Ab: Anti−thyroid peroxidase antibodies, SD: Standard deviation, TSH: Thyroid−stimulating hormone_

Also, demographic characteristics of the participants, including age, sex, weight, height, and body mass index (BMI), were recorded in the relevant form.

For measuring over two-year-old children’s weight, a digital scale made in Iran with a precision of 50 g was used, and for children under two years, a Beurer scale made in Germany with a precision of 5 g was used. They were prepared to sit on the scale with minimal clothing. Furthermore, the height of children under two years was measured while lying down, and the height of children over two years was measured using a vertical meter. The BMI was calculated using the formula BMI = weight (kg)/ height (m^2^). Also, CHD patients were divided according to gender, cyanosis, age, and BMI, and then anti-TTG Ab levels were compared among them. Those ≤ 5 years were compared with those > 5 years only for anti-TTG Ab level, and those with a BMI ≤ 18.5 kg/m^2^ were compared with those with a BMI > 18.5 kg/m^2^.

### Statistical analysis

Data were analyzed using SPSS version 26 software. The data were described in tables and graphs using frequency, percentage, mean, and standard deviation. An independent t-test was used to compare the mean serum anti-TTG Ab values between age groups (≤ 5 years and > 5 years), BMI groups (≤ 18.5 and > 18.5), and cyanotic/non-cyanotic groups. The prevalence was reported as frequency and percentage. A p-value of less than 0.05 was considered significant.

## Results

In this study, 123 CHD patients were evaluated. According to the inclusion criteria, the final analysis was done on 98 patients, of whom 51 (52.04%) were female and 47 (47.96%) were male, with a mean age of 5.32 ± 4.05 years (1–18).

Table 2 shows the demographic characteristics of CHD patients, along with other variables.

**Table 2:** Descriptive characteristics of the study population

Characteristics	Minimum	Maximum	Mean ± SD
Age (year)	1.00	18.00	5.32 ± 4.05
Height (cm)	61.00	179.00	107.22 ± 25.81
Weight (kg)	7.50	84.00	13.78 ± 18.38

Table 3 shows the classification of patients based on cyanosis and Down syndrome, and the classification of patients according to the age of five years and BMI of 18.5 was demonstrated.

**Table 3:** Classifications of congenital heart disease patients

Characteristics	N (%)
**Type of CHD**	
Cyanotic	22 (22.45)
Non-cyanotic	76 (77.55)
**Down syndrome**	
Yes	7 (7.14)
No	91 (92.86)
**Age (years)**	
$\le 5$	66 (67.35)
$>5$	32 (32.65)
**BMI**	
$\le 18.5$	78 (79.59)
$>18.5$	20 (20.41)

_CHD: Congenital heart disease, BMI: Body mass index_

**Fig. 1:**
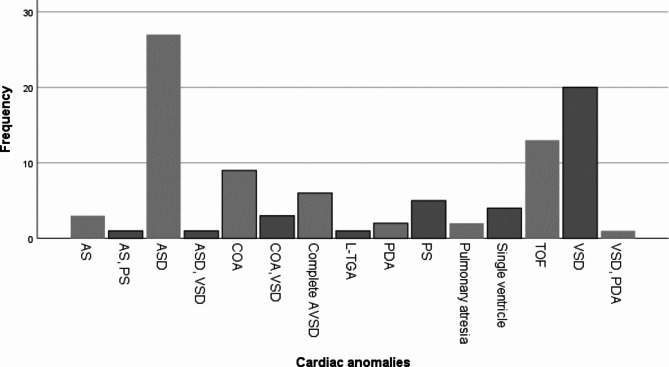
Frequency of different types of cardiac abnormalities

Figure 1 indicates that Atrial Septal Defect (ASD) (27%), Ventricular Septal Defect (VSD) (20%), and Tetralogy of Fallot (TOF) (13%) were the most prevalent CHDs, in sequence.

None of the patients had an anti-TTG Ab serum level above 18 units/mL (Mean ± SD: 3.24 ± 2.69 units/mL, ranging from 0.3 to 16.6 units/mL), and only one girl with VSD-type CHD and BMI of 15.9 had an anti-TTG Ab level of 16.6 units/mL, which was considered as borderline.

In our study, the mean serum anti-TTG Ab did not have a statistically significant difference between boys and girls (p = 0.26). The mean serum anti-TTG Ab was not significantly different between the cyanotic and non-cyanotic groups (p = 0.19). Also, other study variables did not show a statistically significant difference between these two groups (Table 4).

**Table 4:** Serum anti-TTG IgA Ab among patients

Variables	Number	Mean ± SD	P value
**Type of CHD**			
Non- cyanotic patients	76	3.39 ± 2.34	0.192
Cyanotic patients	22	2.44 ± 1.98	
**Gender**			
Female	52	3.25	0.265
Male	46	2.92	
**Age categories**			
≤ 5 years old	66	2.87 ± 2.11	0.460
> 5 years old	32	4.01 ± 3.52	
**BMI categories**			
BMI ≤ 18.5	78	3.16 ± 4.34	3.879
BMI > 18.5	20	3.01 ± 5.12	

In the present study, 100 healthy subjects were examined as the control group for CD. The anti-TTG Ab level in two of them (2%) was between 16 and 18 units/mL, considered borderline.

Also, our study assessed thyroid function among CHD patients. Seven patients had high TSH levels; three were using levothyroxine since early infancy with the diagnosis of congenital hypothyroidism, and parents of the other four were unaware of this issue. Moreover, T4 in these four patients was more than the cut-off points of hypothyroidism, and they had subclinical hypothyroidism. Three of these seven patients had a surgically corrected cyanotic heart disease and were free from cyanosis at the time of the study. One person had Down syndrome with a non-cyanotic CHD. No patients were in critical or ill condition (Table 5). Anti-thyroid peroxidase antibodies were normal in all these seven cases.

**Table 5:** Individual characteristics, heart disorders, and thyroid test results of seven patients with hypothyroidism

Patient	Age/ weight gender	C/ A	Trisomy 21	T4 nmole/L	TSH µU/L	Anti-TPO IU/ml	Aware of disease	On medication
**1**	4.5/ 16.5 F	C	No	7.80	7.40	4.70	No	No
**2**	14.5/ 49 M	C	No	10.70	7.11	3.52	Yes	Yes
**3**	14.0/ 37.50 M	C	No	7.52	25.40	2.3	No	No
**4**	2.42/ 10.55 F	A	No	0.3	12.35	2.64	No	No
**5**	2.00/ 10.50 F	A	Yes	8.35	6.99	3.11	No	No
**6**	1.5/ 9.12 F	A	No	8.43	7.23	2,10	Yes	Yes
**7**	3.50/ 12.13 F	A	No	8.78	7.76	3.64	Yes	Yes

_Anti TPO: anti−thyroid peroxidase antibodies, A: acyanotic congenital heart disease, C: cyanotic congenital heart disease, F: female, M: male, TSH: thyroid stimulating hormone (in the present or past tense)_

Among the 100 people in the control group, two had TSH of more than normal for their age, and their T4 and anti-thyroid peroxidase antibodies levels were also normal, suggesting subclinical non-autoimmune hypothyroidism.

The TSH level of CHD patients and healthy subjects differed significantly (p = 0.01), while the T4 level did not differ significantly between these two groups (p = 0.16).

## Discussion

The prevalence of CD and hypothyroidism varies significantly in different studies among children and adolescents with CHD. The present study examined the frequency of laboratory test abnormalities in children and adolescents with CHD for these two conditions.

As known, CD and hypothyroidism have an essential impact on CHD, and knowing their coexistence can affect the patient’s outcomes.

According to the results of the present study, approximately 1% of the healthy population had borderline anti-TTG Ab levels between 16 and 18 units/mL, and the same percentage was observed in CHD patients. Also, the probability of hypothyroidism was 2% in the healthy population and 7% in CHD patients.

Studies have shown a diverse association between CD and some genetic diseases or chromosomal abnormalities such as CHD, type 1 diabetes mellitus, hypothyroidism, trisomy 21, and Turner syndrome, etc. In some articles, there was no relationship between CD and CHD, or the strength of this relationship was insignificant [18]. However, some studies, including the study of Wingren et al., Mcneish and Anderson, and Kumhar et al., confirmed this relationship [5, 18, 19].

Nevertheless, in our study, no person with CHD had a serum level higher than 18; thus, the CD risk was not higher in patients with CHD than in the general population. According to a study the prevalence of CD seropositivity in Iran was 0.6% [20]. In a review study, the prevalence of CD was higher in Iran than in Western countries (0.6%) [21]. In another study in Iran, the prevalence of CD was 2% in healthy children and adolescents [22].

However, in another Iranian study, the rate of positive celiac serology tests was very high among Down syndrome patients. More than 30% of Down syndrome children with or without CHD had a positive serology test for CD, and the positive rate of the test in individuals with CHD was about 50% [23]. In the other study from Iran, the prevalence of CD was 11% in CHD patients and 3.50% in healthy individuals. This difference was significant and greatly different from global statistics [4].

However, in our study, this prevalence was reported as 1%, and there was no significant difference between the patient and healthy groups. The prevalence of CD in our study is consistent with the global prevalence of this disease in CHD patients. Also, in the present study, no significant difference was observed in the serum level of anti-TTG Ab between cyanotic and non-cyanotic CHD patients. In line with this, one study showed no significant difference between the cyanotic and non-cyanotic groups [4]. According to the present study, there was no significant difference in anti-TTG Ab levels in different age groups (less than and more than five years) and also in two different BMI groups (less than and more than 18.5). In this regard, Taheri et al.‘s study showed that the prevalence of CD in children with failure to thrive was 8.8%, and the mean anti-TTG Ab levels of children did not show a significant difference between age and gender groups [24].

In our study, the prevalence of non-autoimmune hypothyroidism in healthy individuals was 2%, almost equal to that of acquired hypothyroidism in children (3%). However, the prevalence of non-autoimmune hypothyroidism in CHD patients was 7%, three times more than in healthy individuals, and mostly mild and subclinical. People in the control group with high TSH underwent subclinical hypothyroidism, and their T4 level was within the normal range. Parents of four out of seven CHD patients had subclinical hypothyroidism and were unaware of their disease, and three patients were treated with levothyroxine due to previous tests. A study by E. Passeri et al. conducted in Italy also reported an increased risk of about 10 times for congenital hypothyroidism and three times for mild acquired non-immune hypothyroidism among CHD cases [12], which agreed with our study.

Also, other studies reported a higher prevalence of subclinical hypothyroidism in CHD patients, like our study [25]. Notably, tissue-specific thyroid hormone bioactivity is reduced during chronic hypoxia, leading to low T3 syndrome in critically ill patients [9], while our patients were not ill or cyanotic.

### Limitations

This study was limited to the population of Shiraz City. Future studies with larger sample sizes from other cities and provinces of the country are recommended.

## Conclusion

In the present study, serum levels of TSH and non-immune hypothyroidism were higher in patients with CHD than in normal individuals. However, serum anti-TTG Ab levels did not differ between the two groups. Due to the possibility of the negative impact of not diagnosing mild hypothyroidism on the outcome of CHD in children, frequent examination and more attention to thyroid problems in CHD patients is recommended. However, the routine examination of CD tests needs more research.

## Data Availability

The datasets analyzed in this study are available from the corresponding author upon reasonable request.

## Abbreviations

CD: Celiac disease
CHD: Congenital heart disease
TSH: Thyroid stimulating hormone
BMI: Body mass index
ASD: Atrial septal defect
VSD: Ventricular septal defect
TOF: Tetralogy of Fallot
